# A State Censorship Favoured

**Published:** 1917-10-20

**Authors:** 


### THE REPORT OF THE CINEMA COMMISSION.

### A State Censorship Favoured.

The Cinema Commission, which was appointed by the National Council of Public Morals at the instance of certain cinematograph trade associations, has issued its report. The Commission recommends that there should be a conference of representatives of the Home Office, local authorities, the trade, and "a small number of persons who have shown special interest in the picture-house" to draw up model regulations, which should be made statutory; the judgment of the Commission is, moreover, in favour of a State censorship; "the cinema should have the support and the official countenance of the State," it should be placed "in a position of real dignity."

### The Trade Censorship's Limitations.

The conclusions of the Commission, which are founded upon the evidence taken, deal with matters which may be divided broadly into the hygienic and the moral, and are based especially upon the needs arising from the fact that cinema exhibitions are largely frequented by children. It is not proposed to attempt to deal at length here either with the question of the moral welfare of those attending these exhibitions or with the allied question of the quality and freedom from objection of the films exhibited. The cinema, deservedly or not, has attained a place, and is by many highly esteemed, as a form of entertainment. The business grew with extraordinary rapidity, and, not unnaturally, competition caused objectionable features to creep in; much has been done to eliminate these, and every credit must be given to the trade censorship for its share in their elimination. But the trade censorship has its limitations, and from the point of view of good taste room for improvement remains. There is every reason to believe that films of better class than those hitherto too commonly shown would prove attractive not only to those who are now cinema-goers, but to many who have been deterred from attending by their dislike of the American cowboy "melodrama," of the wearisome

THE REPORT OF THE CINEMA COMMISSION—(*continued*).

indoor scenes enacted by grimacing and gesticulating mimes, and of the artistic failure and vulgarity with which the cinema speedily became associated. To have immortalised Charlie Chaplin may be to have added to the gaiety of nations; as a record it is not one of which to be proud. The Commission is assured that the leaders of the trade are not indifferent to "higher considerations," and desire that "such reproach as can be brought against the character of the films shown should be removed as speedily as possible." The popularity of the Ponting Antarctic films is sufficient evidence that public estimation may be attained by legitimate and artistic means.

### Danger in the Picture-House.

The hygienic ideals of the Commission are excellent, if a little vague. A picture-house *should*, of course, be "commodious and well constructed, with ample seating accommodation to avoid the obvious evils of overcrowding"; it should be "thoroughly ventilated and scrupulously clean." Places of entertainment have gradually improved in these respects, and it is to be hoped that they will continue to improve. We have recently dealt with the very difficult question of their effective ventilation in the columns of The Hospital; not altogether, it would appear, to the satisfaction of Mr. F. Fowell, secretary of the Cinematograph Trade Council. We laid stress upon the essential unhealthiness of a place of public assembly from which not only sunlight but even daylight is permanently excluded, and upon the value of vacuum-cleaning apparatus in the attempt to disinfect it. Mr. Fowell, in a letter which we published on September 15, lays claim to highly efficient ventilating arrangements for "most of the recent cinema theatres." He pleads, apparently in extenuation of the fact that it is not usually applied, the insufficiency of the vacuum cleaner to counteract immediately the danger of the person who spits upon the carpet: it can only be said that a carpet is an unsuitable floor covering where patrons are in the habit of so spitting, and that the danger of infected sputum becomes potent on drying, when a vacuum cleaner is the one and only means (in the case of a carpet) of removing the danger.

### The Prevention of Eye-Strain.

Certain conclusions were arrived at by the Commission in connection with the well-known cinema eye-strain. One of the most interesting is that "a new copy of each film should be provided at every performance": that this is deemed to be an unattainable ideal is shown by the addition of the words "or at least at frequent intervals." The object of this, and of some other suggestions, is evidently to reduce, as far as possible, the flicker of the pictures and the flashing effect which is generally the result of abrasions of the sensitised coating of the film. No number of new copies will, however, atone for defects in the negative. The Commission seems to have little hope of the provision of multiple new copies; "some of the reforms may prove so costly that it would be impossible to continue to provide popular entertainment as cheaply as heretofore." The suggestion that children should sit at a distance from the screen not less than one and a half times its own height is a reasonable one: that they should not attend the cinema house "too frequently," or remain "too late," or stay "too long at any one visit" are facts sufficiently obvious upon the accepted principle that they should not have too much of a good thing—which the Commission seems to acknowledge the cinema to be. There should, however, be power to close these resorts to children in times of epidemic, and this should be promptly and freely exercised. The futile anomaly of keeping such places open whilst elementary and Sunday schools are closed should cease forthwith; this is one of the things which are better ordered in America.

### What Legislation Should Aim at.

The Commission "agreed that legislation which had the effect of placing" the cinema as a place of entertainment "beyond the reach of the million should be very slowly undertaken." We will go further: there should be no such legislation, hasty or otherwise. "The million" wants the cinema and should be allowed to have it. Legislation should have the aim and object of elevating it, and should effect such improvements and reforms as will ensure the physical and moral well-being of the million in its enjoyment of it.

